# Supplementary material for: Global probabilistic projections of extreme sea levels show intensification of coastal flood hazard
Source: Nat Commun. 2018 Jun 18;9:2360. doi: 10.1038/s41467-018-04692-w (PMC6006144; doi:10.1038/s41467-018-04692-w)
Supplement: Supplementary file 1 — Supplementary Information [file 41467_2018_4692_MOESM1_ESM.pdf]

Probabilistic projections of global extreme sea levels show intensification of coastal flood hazard: Supplementary Information

Supplementary Tables

Supplementary Table 1. Projections of changes in ESLs along different global regions: Changes in ESL under RCP4.5 and RCP8.5 for 2050 and 2100 (in cm). The global coastline is divided in 14 geographical regions in order to better reflect the spatial variations of changes in ESL, and median values are provided along with very likely range (90% probability) given in brackets.

	**RCP4.5**		**RCP8.5**	
	2050	2100	2050	2100
**WEST N. AMERICA**	26 [19-34]	53 [37-71]	36 [30-43]	90 [64-167]
**GULF OF MEXICO**	22 [11-34]	52 [29-78]	31 [22-44]	86 [54-188]
**EAST N. AMERICA**	26 [16-36]	53 [34-73]	33 [25-41]	82 [54-161]
**WEST S. AMERICA**	25 [18-32]	59 [41-80]	33 [27-39]	92 [66-166]
**EAST S. AMERICA**	24 [18-29]	57 [42-75]	30 [25-36]	91 [65-164]
**NORTH EUROPE**	23 [13-34]	46 [28-66]	29 [21-39]	78 [51-157]
**MEDITERRANEAN SEA**	19 [10-28]	46 [25-71]	27 [20-40]	76 [47-175]
**WEST AFRICA**	25 [21-29]	56 [40-74]	31 [27-39]	89 [64-181]
**EAST AFRICA**	20 [14-27]	52 [35-71]	28 [23-36]	87 [61-175]
**NORTH INDIAN OCEAN**	19 [12-26]	50 [34-70]	27 [22-36]	86 [59-171]
**AUSTRALIA**	23 [11-36]	55 [34-79]	32 [24-42]	93 [61-179]
**SOUTH EAST ASIA**	22 [15-31]	56 [37-79]	30 [24-39]	91 [62-188]
**NORTH EAST ASIA**	21 [9-33]	52 [32-73]	25 [17-35]	79 [51-160]
**SOUTH PACIFIC**	21 [7-36]	57 [29-88]	32 [24-43]	100 [67-203]
**WORLD**	**24 [14-34]**	**54 [34-76]**	**31 [24-41]**	**86 [58-172]**

Supplementary Table 2. Projections of SLR along different global regions: *SLR under RCP4.5 and RCP8.5 for 2050 and 2100 (in cm). The global coastline is divided in 14 geographical regions in order to better reflect the spatial variations of changes in ESL, and median values are provided along with very likely range (90% probability) given in brackets.*

	**RCP4.5**		**RCP8.5**	
	2050	2100	2050	2100
**WEST N. AMERICA**	23 [12-36]	49 [19-81]	30 [18-51]	83 [44-195]
**GULF OF MEXICO**	24 [14-35]	54 [25-87]	30 [20-51]	86 [50-203]
**EAST N. AMERICA**	24 [13-37]	49 [20-80]	31 [18-52]	82 [43-194]
**WEST S. AMERICA**	23 [11-35]	54 [25-89]	29 [18-48]	88 [50-191]
**EAST S. AMERICA**	24 [13-36]	56 [26-91]	30 [19-49]	89 [51-197]
**EUROPE**	20 [8-32]	41 [11-72]	27 [14-47]	75 [36-184]
**MEDITERRANEAN SEA**	21 [11-31]	48 [21-77]	27 [16-46]	77 [43-187]
**WEST AFRICA**	25 [14-36]	56 [27-89]	31 [21-52]	89 [53-206]
**EAST AFRICA**	23 [13-34]	54 [25-86]	29 [19-50]	88 [51-202]
**NORTH INDIAN OCEAN**	21 [11-32]	52 [23-84]	27 [17-48]	85 [49-201]
**AUSTRALIA**	24 [13-36]	57 [25-93]	30 [20-51]	92 [53-206]
**SOUTH EAST ASIA**	24 [12-35]	57 [26-93]	30 [19-52]	91 [52-214]
**NORTH EAST ASIA**	21 [10-32]	51 [21-83]	27 [17-48]	83 [47-198]
**SOUTH PACIFIC**	25 [13-38]	59 [27-97]	31 [20-54]	95 [54-217]
**TOTAL**	**23 [12-35]**	**51 [21-84]**	**30 [18-50]**	**85 [47-198]**

Supplementary Table 3. Projections of changes in ESL contributions from climate extremes along different global regions (η_{CE}): *Changes in η_{CE} under RCP4.5 and RCP8.5 for 2050 and 2100 (in cm). The values express averages along 14 geographical regions defined on the basis of geographical proximity, as well as similarities in metocean, atmospheric conditions, and η_{CE} trends. Median values are provided along with likely range (67% probability) given in brackets.*

	**RCP4.5**		**RCP8.5**	
	2050	2100	2050	2100
**NEWFOUNDLAND & LABRADOR**	3 [-7-14]	5 [-4-15]	4 [-3-12]	8 [5-11]
**WEST N ATLANTIC**	1 [-10-13]	4 [-11-18]	6 [-4-18]	7 [3-12]
**CENTRAL AMERICA**	-6 [-12--0]	-7 [-11--1]	-4 [-4--3]	-5 [-7--3]
**SOUTH S. AMERICA**	0 [-8-8]	5 [-7-14]	-3 [-6-0]	-9 [-10--7]
**SOUTHERN OCEAN**	10 [6-14]	9 [4-14]	12 [9-16]	19 [16-22]
**NORTH EUROPE**	2 [0-4]	4 [-2-10]	11 [4-19]	17 [8-25]
**S EUROPE & NW AFRICA**	3 [1-5]	4 [0-8]	-2 [-2--1]	-4 [-5--2]
**E AFRICA**	-11 [-17--4]	-18 [-25--10]	-12 [-16--6]	-21 [-32--10]
**S & SE ASIA, AUSTRALIA**	-5 [-29-25]	-3 [-30-29]	-5 [-19-11]	-22 [-43-2]
**E CHINA & JAPAN SEA**	17 [-26-65]	36 [-6-82]	10 [-0-21]	27 [11-42]
**OKHOTSK & W BERING**	3 [-17-28]	-1 [-13-13]	-11 [-20--3]	-15 [-25--7]
**ALASKA-E BERING**	4 [-0-9]	5 [1-9]	4 [1-7]	11 [6-16]
**S PACIFIC**	-6 [-19-8]	-5 [-14-5]	-0 [-4-5]	10 [-1-20]

Supplementary Figures

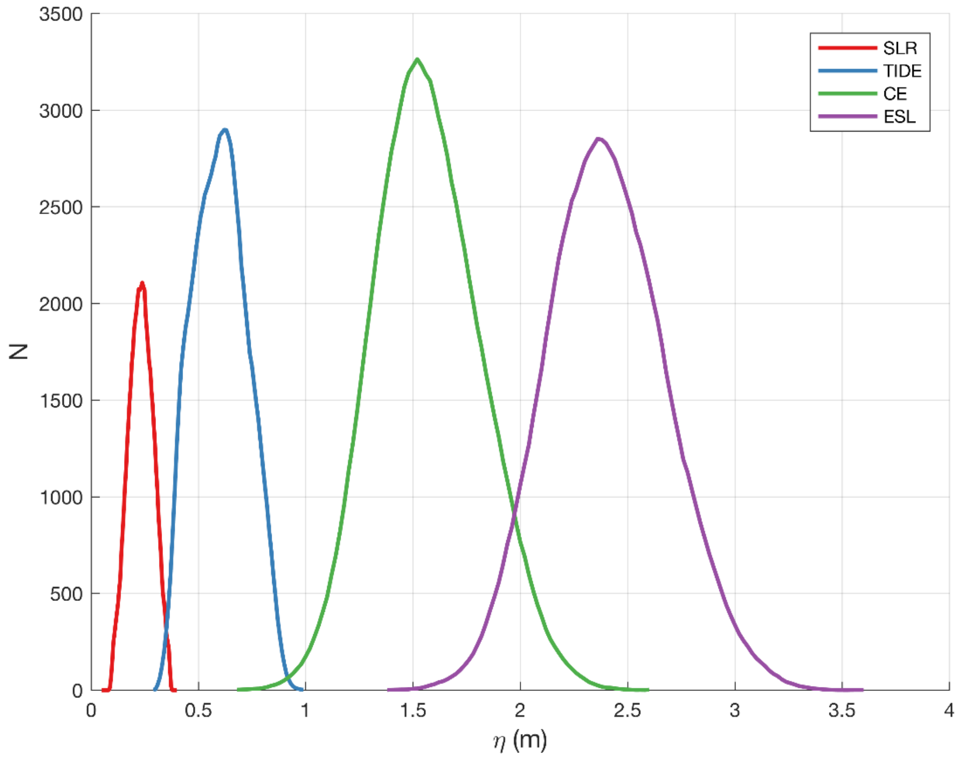

Supplementary Figure 1. Monte Carlo approach example *applied to generate ESL PDFs (purple), from the combination of the PDFs of Sea Level Rise (red), high tide water levels (blue), water levels from storm surges and waves (green).*

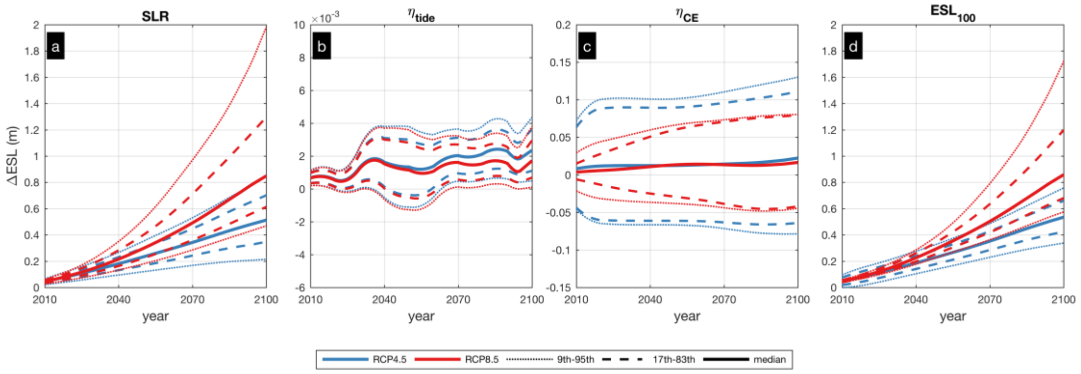

Supplementary Figure 2. Projected changes in global ESL components: *Mean Sea Level (SLR; a); mean high tide water level (η_{tide}; b); 100-year water level from waves and storm surge (η_{CE}; c); and their combined 100-year Extreme Sea Level ESL (d), under RCP4.5 (blue) and RCP8.5 (red). Heavy: median, dotted: 5th-95th percentiles and dashed: 17th-83th percentiles.*

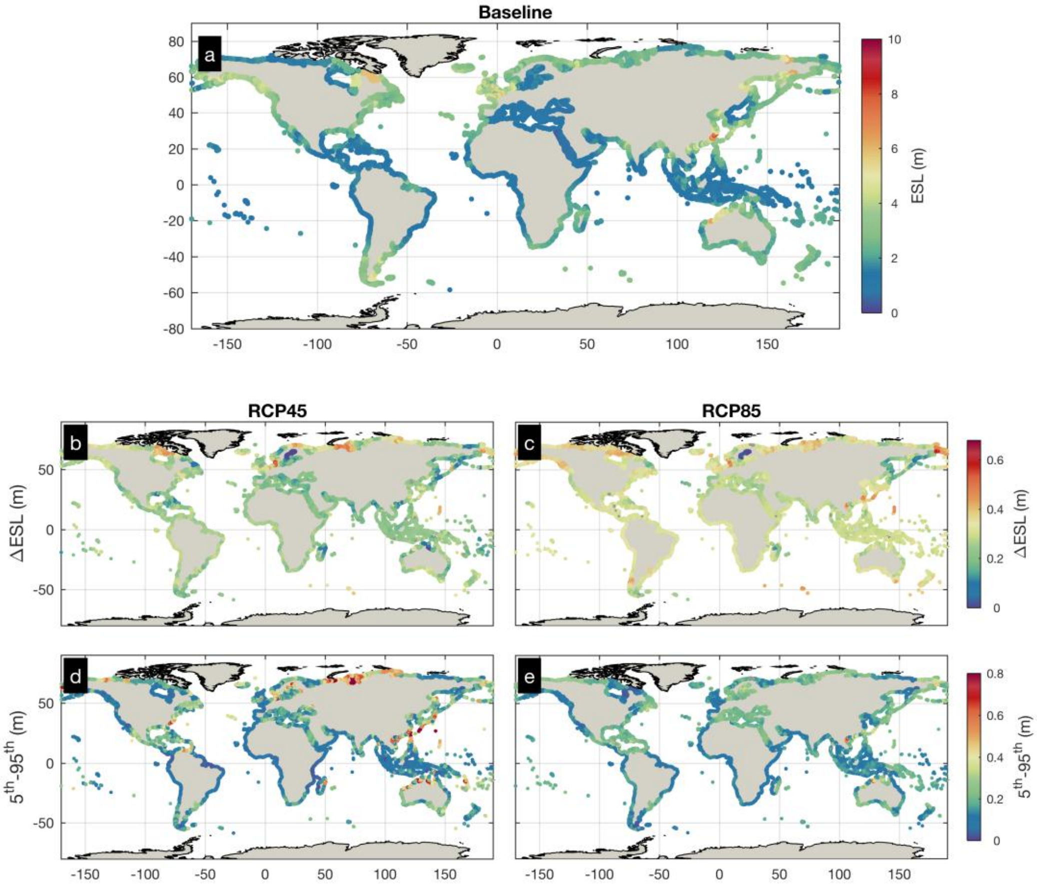

Supplementary Figure 3. Present global ESLs, changes in view of climate change and uncertainty: *Maps show the median present-day 100-year ESL (a) and the projected changes in ESL_{100} expressed by the median and the very likely range under RCP4.5 (b, d) and RCP8.5 (c, e) by 2050.*

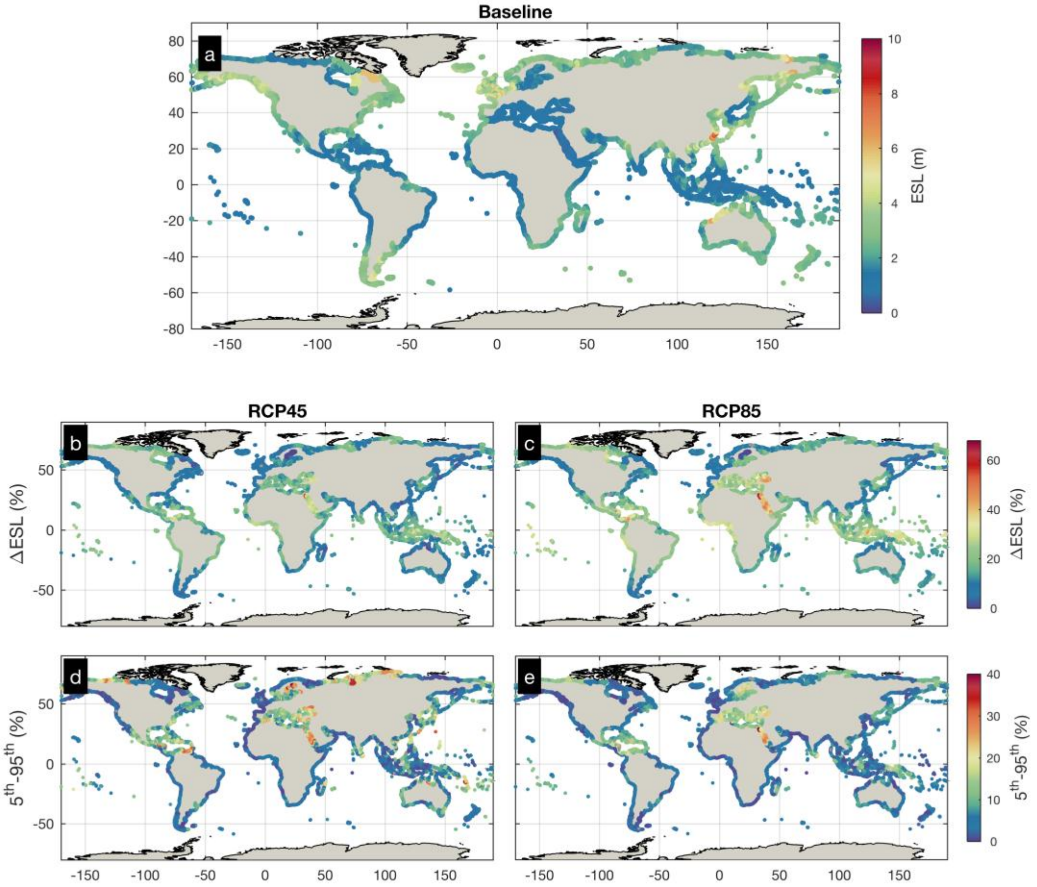

Supplementary Figure 4. Present global ESLs, relative changes in view of climate change and uncertainty*: Maps show the median present-day 100-year ESL (a) and the projected relative changes in ESL_{100} expressed by the median and the very likely range under RCP4.5 (b, d) and RCP8.5 (c, e) by 2050.*

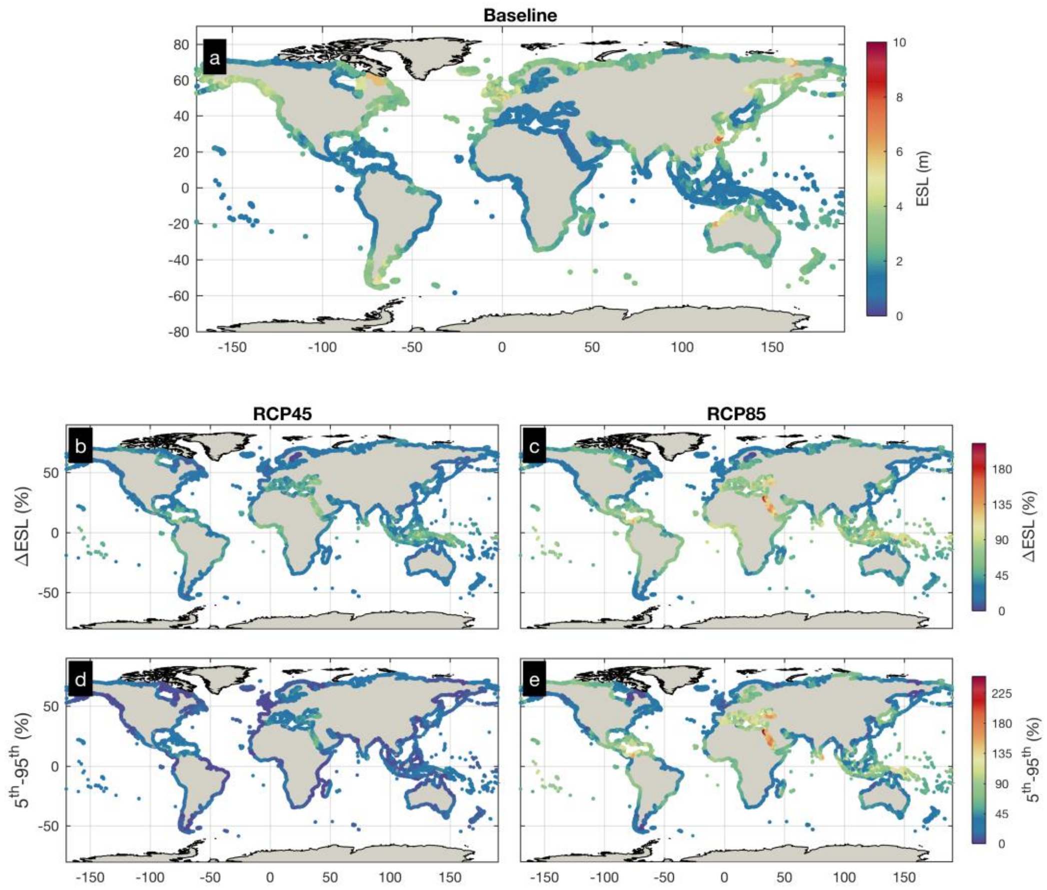

Supplementary Figure 5. Present global ESLs, relative changes in view of climate change and uncertainty*: Maps show the median present-day 100-year ESL (a) and the projected relative changes in ESL_{100} expressed by the median and the very likely range under RCP4.5 (b, d) and RCP8.5 (c, e) by 2100.*

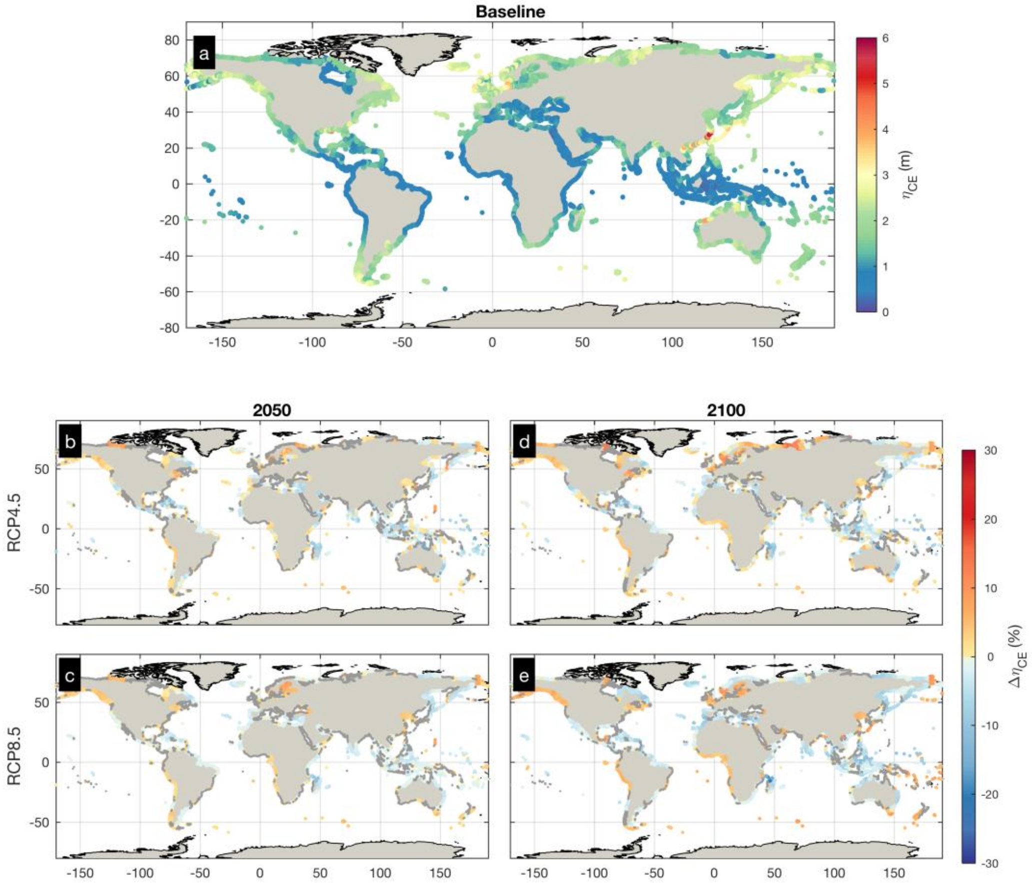

Supplementary Figure 6. Present contributions of climate extremes to global ESLs (η_{CE}) and projected relative changes: *Maps show the median present-day 100-year η_{CE} (a) and projected relative changes (%$\Delta\eta_{CE}$) under RCP4.5 by 2050 (b) and 2100 (c), and under RCP8.5 by 2050 (d) and 2100 (e). Warm/cold colors express an increase/decrease, respectively, while points with high uncertainty are shown in grey (|CV|>1).*

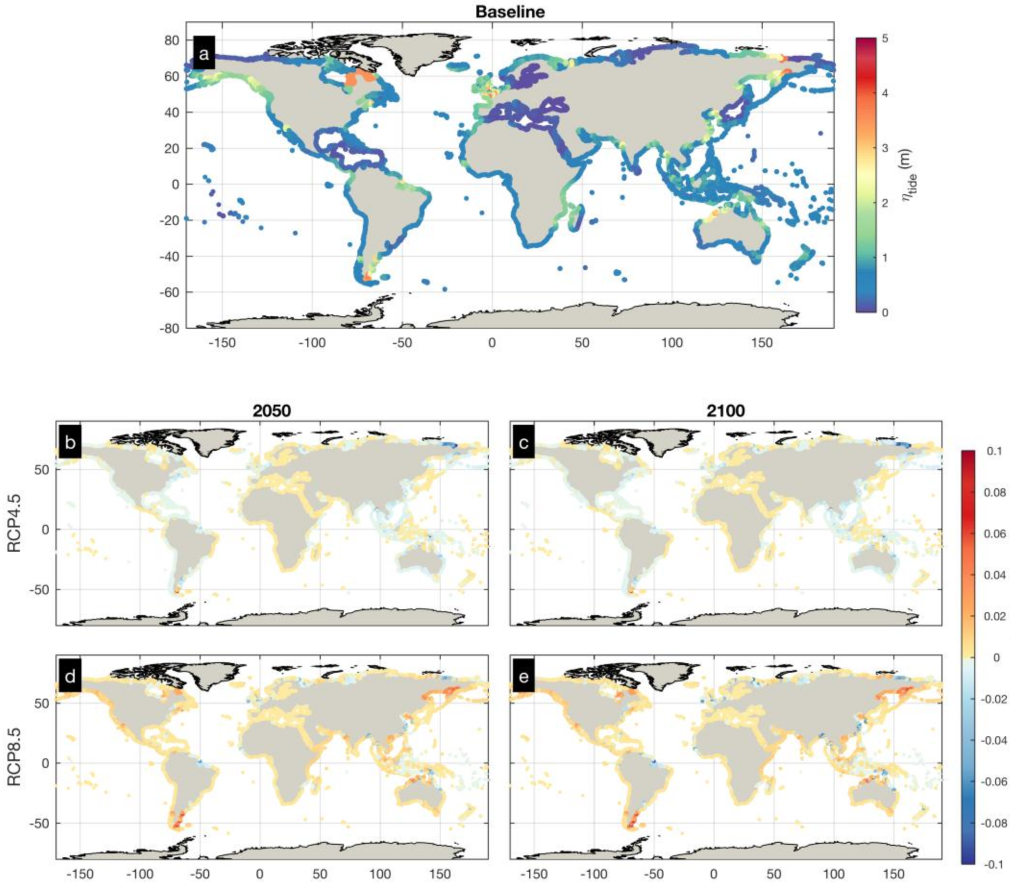

***Supplementary Figure 7*. Global high tide water levels (η_{tide}) and projected changes:** *Maps show the median present-day η_{tide} (a) and projected changes under RCP4.5 by 2050 (b) and 2100 (c), and under RCP8.5 by 2050 (d) and 2100 (e). Warm/cold colors express an increase/decrease, respectively, while points with high uncertainty are shown in grey (|CV|>1).*

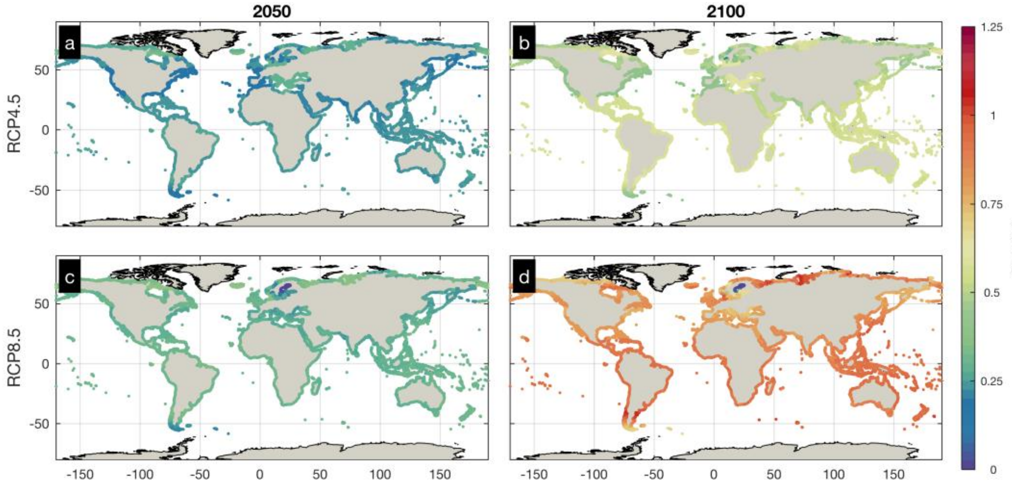

***Supplementary Figure 8*. SLR projections along the global coastline:** *Maps show the median projected increase in MSL under RCP4.5 by 2050 (a) and 2100 (b), and under RCP8.5 by 2050 (c) and 2100 (d).*

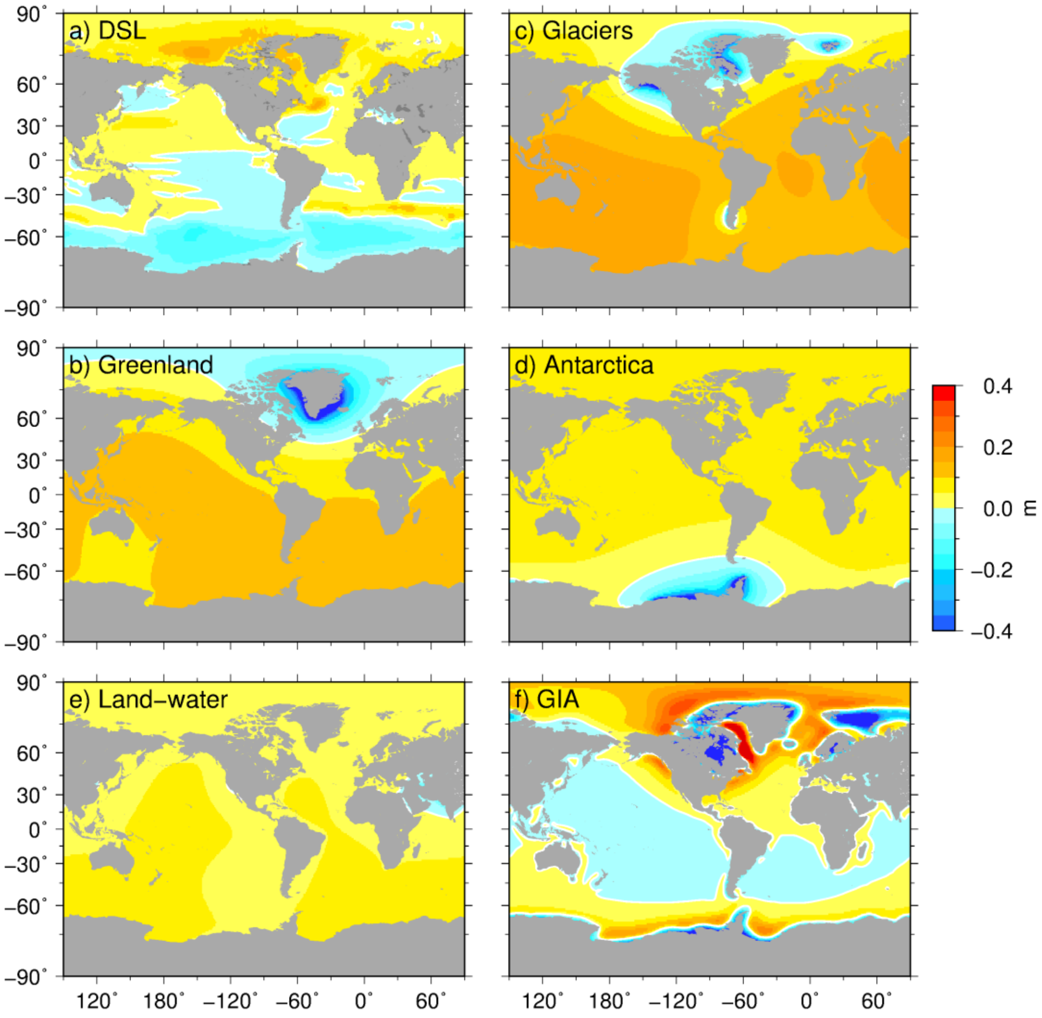

Supplementary Figure *9. Median projected relative sea-level change by 2100 (relative to 1985-2006) for RCP4.5 of sea-level components, a) dynamic sea-level, b) Greenland, c) Glaciers, d) Antarctica, e) Land-water and f) glacial isostatic adjustment. The global average steric sea-level change is used in addition to these components (+0.20 m by 2100).*

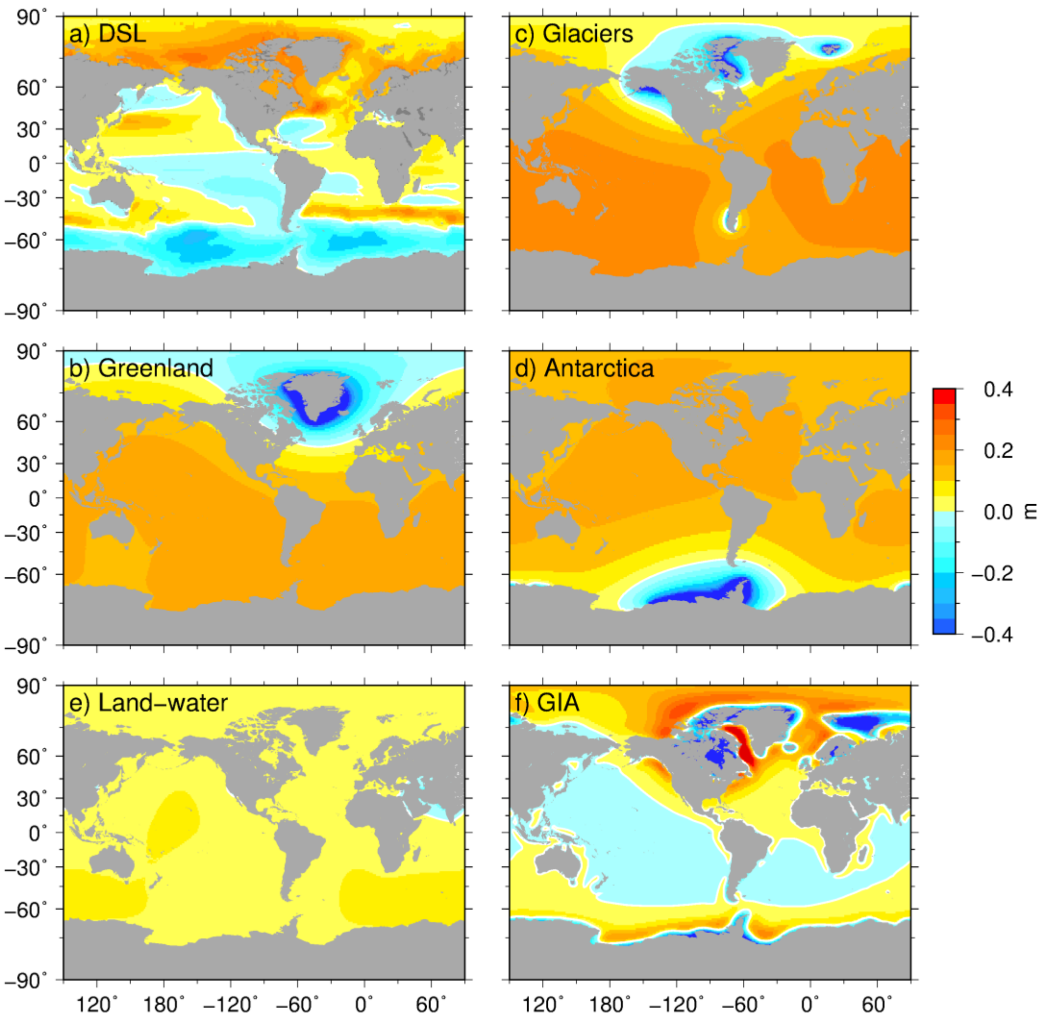

Supplementary Figure *10. Median projected relative sea-level change by 2100 (relative to 1985-2006) for RCP8.5 (see text for scenario details) of sea-level components, a) dynamic sea-level, b) Greenland, c) Glaciers, d) Antarctica, e) Land-water and f) glacial isostatic adjustment. The global average steric sea-level change is used in addition to these components (+0.32 m by 2100).*

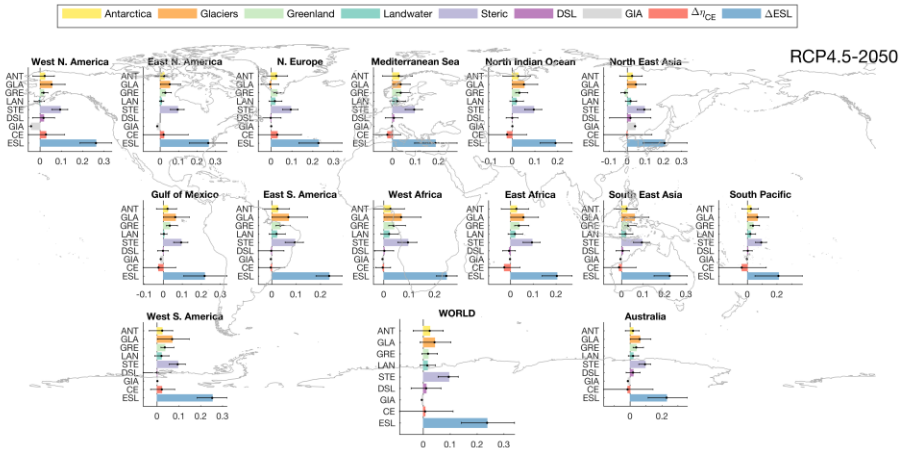

Supplementary Figure 11*. *Break-down of projected changes in ESL components, under RCP4.5 in 2050. *Projected changes in ESL contributions from Antarctica (ANT), glaciers (GLA), Greenland (GRE),*

land-water (LAN), steric-effects (STE), dynamic sea level (DSL), glacial isostatic adjustment (GIA), climate extremes (CE), as well as the combined 100-year ESL. Bars express the median values, black error lines the 5% and 95% quantiles. Values shown are expressed in m and reflect spatial averages for 14 regions and worldwide.

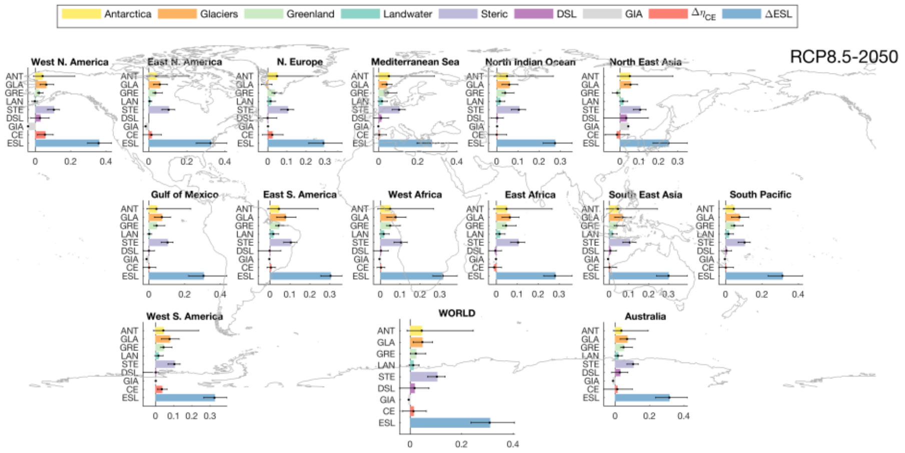

***Supplementary Figure 12**. **Break-down of projected changes in ESL components, under RCP8.5 in 2050.** Projected changes in ESL contributions from Antarctica (ANT), glaciers (GLA), Greenland (GRE), land-water (LAN), steric-effects (STE), dynamic sea level (DSL), glacial isostatic adjustment (GIA), climate extremes (CE), as well as the combined 100-year ESL. Bars express the median values, black error lines the 5% and 95% quantiles. Values shown are expressed in m and reflect spatial averages for 14 regions and worldwide.*
